# A multidisciplinary approach to the functional abnormalities of the migrainous brain and non-invasive interventions to treat them

**DOI:** 10.1186/1129-2377-14-S1-I5

**Published:** 2013-02-21

**Authors:** Alessandro Viganò, Volodymyr B Bogdanov, Jean Schoenen

**Affiliations:** 1 Headache Research Unit. Dept of Neurology, University of Liège, Belgium; 2 Brain Morphometry and Dynamics Lab., Dept of Anatomy, Histology, Forensic Medicine and Orthopaedics, Sapienza University of Rome, Italy; 3 Neurology A. Dept of Neurology and Psichiatry. Sapienza University of Rome, Italy; 4 Department of Neurology, University of Utah, Salt Lake City, USA; 5 Giga-Neurosciences, University of Liège, Belgium

## Background

Migraine is characterized pathophysiologically by an interictal habituation deficit of information processing, responsible for cortical hyperresponsiveness, which normalizes during attacks [1]. Furthermore, descending pain control systems (DPCSs) can be impaired in episodic and chronic migraine [3,4]. Noninvasive neuromodulation of the cerebral cortex may be effective in migraine prophylaxis [2] including, in chronic migraine, modulation of the dorso-lateral prefrontal cortex (DLPFC) that is part of the DPCSs [5].

## Aims

To identify targets and neuromodulation interventions for migraine treatment by exploring the role of DLPFC in heterotopic noxious analgesia and by using transcranial magnetic (rTMS) and direct current stimulation (tDCS).

## Methods

To this purpose, we performed the following studies:

1) we studied the modification of visual cortex (VC) responsiveness, as assessed by visual evoked potentials (VEPs), induced by excitatory intermittent theta-burst rTMS (i-TBS) [6] or anodal (i.e. excitatory) tDCS (AtDCS) [7] in healthy volunteers (HV) and in episodic migraineurs (EM)

2) we used AtDCS on VC (twice/week for 8 weeks) in a pilot study of migraine prevention.

3) we used fMRI to explore the role of DLPFC in heterotopic noxious analgesia induced by painful cold in EM during and between attacks.

## Results

i-TBS induced a sustained increase of 1st block VEP amplitude and habituation in HV (n=13). AtDCS increased habituation in HV (n=11) and EM (n=12).

In the prophylactic trial (n=7), AtDCS reduced significantly migraine frequency (-40%), and attack duration (-43.25%).

In migraineurs, interictally, cold-induced analgesia, which was related to baseline autonomic arousal, was proportional to cold-induced BOLD responses in the DLPFC. During an attack, BOLD responses induced in the premotor cortex by cold application on the foot were also significantly increased.

## Conclusions

Excitatory neuromodulation (both i-TBS or tDCS) of the visual cortex in HV induces changes in cortical responsiveness that should be able to normalize the interictal abnormalities found in migraineurs. Our pilot therapeutic study suggests that excitatory anodal tDCS could have a preventive anti-migraine effect. Finally, the impaired descending analgesia that characterizes migraine could be amenable to AtDCS or i-TBS neuromodulation targeting the DLPFC and premotor cortices.
